# T Lymphocyte Infiltration in Association with IDO1 Expression in Resected Lung Adenocarcinoma and Normal Adjacent Lung Tissues

**DOI:** 10.1155/2022/2381018

**Published:** 2022-02-10

**Authors:** Xiaoling Zhao, Yaran Li, Xu Yang, Xiaochong Zhang, Jing Xie, Shaoteng Li, Hongzhen Liu, Jun Guo, Lili He, Wei Chen, Dengxiang Liu

**Affiliations:** ^1^Cancer Laboratory, Xingtai People's Hospital, Xingtai, Hebei, China; ^2^Department of Endocrinology, Xingtai People's Hospital, Xingtai, Hebei, China; ^3^Personnel Department, Xingtai People's Hospital, Xingtai, Hebei, China; ^4^Department of Science and Education, Xingtai People's Hospital, Xingtai, Hebei, China; ^5^Department of Anesthesiology, Xingtai People's Hospital, Xingtai, Hebei, China; ^6^CT Department, Xingtai People's Hospital, Xingtai, Hebei, China; ^7^Xingtai Health Commission, Xingtai, Hebei, China; ^8^Department of Oncology, Xingtai People's Hospital, Xingtai, Hebei, China; ^9^Department of Radiology, Xingtai People's Hospital, Xingtai, Hebei, China; ^10^Administration Office, Xingtai People's Hospital, Xingtai, Hebei, China

## Abstract

**Background:**

Indoleamine 2,3-dioxygenase 1 (IDO1) catalyzes the first step of tryptophan catabolism in the kynurenine (Kyn) pathway. IDO1 downregulates natural killer cell receptors, and by mechanism, tumor cells escape immune surveillance.

**Methods:**

IDO1 protein and mRNA were assessed by immunohistochemistry, immunoblotting, and PCR in the 68 resected lung adenocarcinomas at stages I–III as well as adjacent normal lung tissues. Infiltration of CD3, CD8, and CD4 lymphocytes in the tumor and adjacent normal lung tissues was assessed by immunohistochemical staining.

**Results:**

IDO1 protein and mRNA were detected in various stages of lung adenocarcinoma with highest expression at stage III. In contrast, biomarkers of T cell subset, CD3, CD4, and CD8, were highly expressed in the normal lung tissues and stage I adenocarcinoma tissues but significantly reduced in the stage II and III tumor tissues.

**Conclusions:**

The current study demonstrated that the higher level of IDO1 expression in the lung adenocarcinoma was, the less infiltration of T lymphocytes was found in the tumors. Findings of this study indicated that IDO1 may contribute to the reduction of T lymphocyte infiltration into the lung adenocarcinoma.

## 1. Introduction

Lung cancer is the predominant cancer in China and one of the most commonly seen malignant tumors in the world [1]. Lung cancer is the second most common cancer in men, after prostate cancer, and the second most common cancer in women, after breast cancer [2]. According to the characteristics of cell origin and differentiation, lung cancer is classified as small-cell lung cancer (SCLC) and non-small-cell lung cancer (NSCLC). The NSCLC is further classified into adenocarcinoma (ADC), squamous cell carcinoma (SQC), and large cell carcinoma (LCC). SCLC and NSCLC accounted for 15% and 85%, respectively, and adenocarcinoma accounted for 40% of lung cancer [3, 4].

Lung adenocarcinoma is more likely to develop in women and those without a history of smoking, in which some targetable gene mutations including growth factor receptor (EGFR), anaplastic lymphoma kinase (ALK), BRAF, and ROS1 were positively expressed [5–8]. While lung adenocarcinoma with gene mutation can be treated with targeted immunotherapy, tumor cells often acquire mutation-driven drug resistance within 9–12 months after chemotherapy and often escape immune attack through enhancing immunosuppressive mechanisms, such as coregulation of ligands/receptors and thyroxine [9]. Targeting immune checkpoint factors, for instance, the programmed cell death-1 (PD-1)/programmed cell death-ligand 1 (PD-L1) pathway, has emerged as a durable and prominent therapeutic option in diverse malignancies including NSCLC [10–12]. However, most of the patients who initially responded to immunotherapy eventually acquired resistance, and the disease process returned to a natural state. While the mechanisms of innate and acquired resistance to immunotherapy in various types of malignancies are not fully understood, it may be associated with insufficient antitumor T cell generation, inadequate antitumor T cell effector function, impaired formation of T cell memory, lack of tumor antigens, or upregulation of other immune checkpoints [13, 14].

Indoleamine 2,3-dioxygenase 1 (IDO1) is a tryptophan catabolic enzyme that catalyzes the first and rate-limiting step of tryptophan catabolism in the kynurenine (Kyn) pathway [15]. The toxic Kyn metabolites can indirectly suppress effector T cell response by favoring differentiation of regulatory T cells (Treg) [16–18]. IDO1 promotes immune escape through downregulating natural killer cell receptors, and by mechanism, the tumor cells escape immune surveillance [16, 19]. In addition, IDO1 overexpression is associated with poor prognosis in many malignant tumors [20, 21]. However, to our knowledge, expression of IDO1 in various clinical stages of lung adenocarcinoma and its association with tumor-infiltrating T lymphocyte subsets has not been reported. Therefore, the current study was designed to assess the expression of IDO1 and biomarkers of T cell subsets (CD3, CD4, and CD8) in the lung adenocarcinomas as well as adjacent normal lung tissues, which were resected from the patients at stage I, II, and III lung adenocarcinoma.

## 2. Materials and Methods

### 2.1. Patients and Resected Lung Tissues

Lung tissue samples from 68 patients who had received surgical resection of primary lung adenocarcinoma from May 2017 to February 2021 at the Department of Thoracic Surgery, Xingtai People's Hospital, were used in the current study. The following specimens were collected and used in this study: 68 cancer tissue specimens (40 cases of stage I, 13 cases of stage II, and 15 cases of stage III lung adenocarcinoma tissues) and 20 normal tissues adjacent to the cancer (10 cases of paraffin embedded and 10 cases of fresh tissue). None of the patients have received chemotherapy, radiotherapy, or targeted immunotherapy before enrolling into this study. This study was approved by the Institutional Review Board of Xingtai People's Hospital (IRB No. 2020-084), and informed consents were obtained from the patients.

### 2.2. Histology and Immunohistochemistry

A total of 78 formalin-fixed, paraffin-embedded (FFPE) tissues (68 lung adenocarcinoma tissue specimens and 10 adjacent lung tissues) were obtained from 68 patients for immunohistochemical staining following routine protocol. Briefly, the FFPE tissues were sliced into sections at a thickness of 4 *μ*m and roasted at 75°C followed by blocking with 2% normal goat serum. The specimens were then incubated with the following rabbit or mouse monoclonal primary antibodies at 4°C overnight: anti-IDO1, anti-CD3, anti-CD4, and anti-CD8 (all antibodies were purchased from Abcam, Shanghai, China). After washing, HRP-conjugated anti-rabbit or anti-mouse antibodies were applied for 1 h at room temperature. Staining was visualized with DAB substrate followed by counterstaining with hematoxylin.

### 2.3. Western Blotting

The tissues were grounded to a solid-free condition. After centrifugation, the supernatant was transferred to a new tube for protein extraction. Electrophoresis on 4–12% Bis-Tris gels with 3-(N-morpholino)-propane sulfonic acid running buffer (Invitrogen, Paisley, UK) was performed in an X Cell II Mini-Cell electrophoresis unit (Invitrogen). The proteins were transferred onto a nitrocellulose membrane followed by blocking with 10% milk/0.1% Triton-X-100/PBS. Subsequently, the membranes were incubated with the primary antibodies (IDO1: dilution 1 : 200, Santa Cruz Biotechnology, Santa Cruz, CA, USA), followed by horseradish peroxidase-conjugated secondary antibodies (anti-rabbit IgG for IDO, Santa Cruz Biotechnology). Immunoreactive proteins were visualized using the biochemiluminescence technique and Hyperfilm ECL (GE Healthcare, Uppsala, Sweden) development.

### 2.4. Real-Time RT-PCR

Forty fresh tissue samples were used to extract total RNA, which was extracted with QIAzol reagent (Qiagen, Valencia, CA) and reverse transcribed into cDNA using ReverTra Ace reverse-transcription kit (TOYOBO Co., Osaka, Japan). SYBR/ROX Master Mix was used for real-time PCR. The primers were as follows: IDO1: forward: TGCTTGGAGAAAGCCCTTCA; IDO1: reverse: ACCCTTCATACACCAGACCG. Level of IDO1 gene expression was expressed by the ratio of cDNA relative to the EEF1a1 as endogenous control using the 2^−ΔΔ^Ct method. Real-time PCR was performed in triplicate and repeated at least three times in separate experiments.

### 2.5. Statistical Analysis

Statistical analysis was performed using GraphPad Prism 5 (GraphPad Software, Inc, San Diego, CA, USA). Pearson's *χ*^2^ test was used to compare categorical data. Student's *t*-test was used to determine significance of the means of two sets of data. A *P* value < 0.05 was considered statistically significant.

## 3. Results

Patients' cohort and clinical characteristics are shown in Table 1. Patients' average age was 60.95 years old (range 32–76); 57.35% (39/68) patients were male, and 42.65% (29/68) were female; 26.47% (18/68) patients were current smokers, and 7.35% (5/68) were ex-smokers. By the criteria of the 8th edition for cancer staging by the American Joint Committee on Cancer, 58.82% (40/68) cases were stage I, 19.12% (13/68) cases were stage II, and 22.06% (15/68) cases were stage III lung adenocarcinoma. Patients at stage IV were not enrolled into this study because they were rarely eligible for surgery.

**Figure 1:**
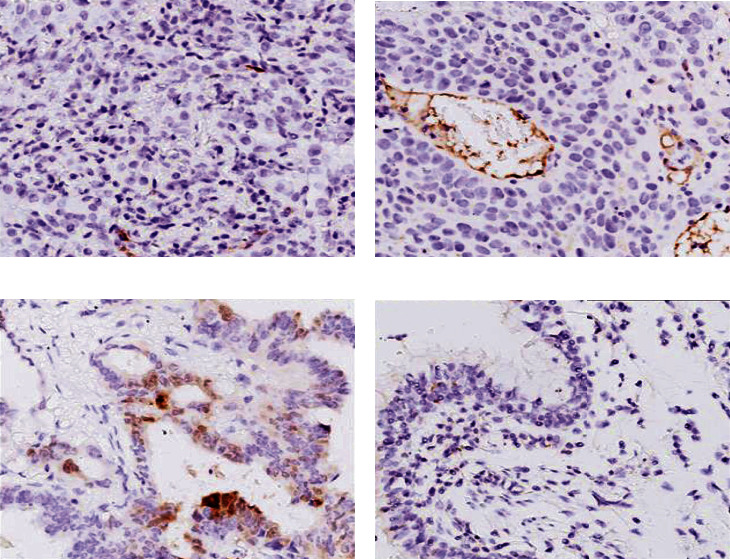
Expression of IDO1 in the lung adenocarcinoma and adjacent normal tissues. IDO1 expression in the resected lung adenocarcinoma and adjacent normal tissues was assessed by immunohistochemical staining as described in the methods. (a) Stage I lung adenocarcinoma. (b) Stage II lung adenocarcinoma. (c) Stage III lung adenocarcinoma. (d) Adjacent normal lung tissue. Magnification: 400x.

**Figure 2:**
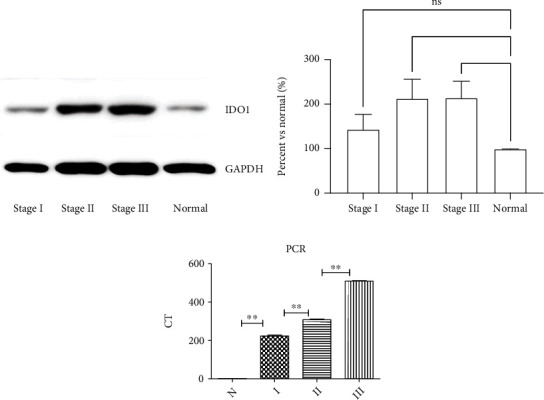
Protein and mRNA level of IDO1 in the lung adenocarcinoma and adjacent normal tissues. (a) Representative image data of IDO1. Protein level of IDO1 in the lung adenocarcinoma and adjacent normal lung tissues was assessed by immunoblotting as described in the methods. GAPDH was used as loading control. (b) Semiquantification of the immunoblotting data. Image was analyzed, and density of each band was obtained by the Image J software. Data was an average of three immunoblotting bands obtained in three separate experiments. GAPDH was used as internal control, and data was expressed as percent versus normal (%). (c) Expression of IDO1 mRNA. Quantification of IDO1 mRNA was performed by real-time RT-PCR as described in the method. *N*: adjacent normal lung tissue. I, II, and III: stage I, II, and III adenocarcinoma. ^∗∗^*P* < 0.01.

Evaluation on the IDO1 level by immunohistochemical staining showed that heterogeneity existed in the lung adenocarcinomas. As shown in Figure 1, IDO1 was not detectable in the adjacent normal lung tissues (Figure 1(d)), was barely detectable in stage I lung adenocarcinomas (Figure 1(a)), easily detectable in stage II tumor tissues (Figure 1(b)), and most highly expressed in stage III tumor tissues (Figure 1(c)). Specifically, as shown in Table 2, expression of IDO1 was low in majority (92.5%, 37/40) of stage I lung adenocarcinomas, with only 7.5% (3/40) of stage I tissues showed highly expressed IDO1 (*P* < 0.01). In contrast, high expression of IDO1 was detected in majority (93.33%, 14/15) of stage III lung adenocarcinomas, with only 6.7% (1/15) of stage III tumor tissues showed low expression of IDO1 (*P* < 0.01). Consistently, immunoblotting and real-time RT-PCR results showed that both protein (Figures 2(a) and 2(b)) and mRNA expression (Figure 2(c)) of IDO1 were significantly higher in the tumor tissues of stages II and III compared to that in normal lung tissues, and that it was highest in stage III tumors.

**Figure 3:**
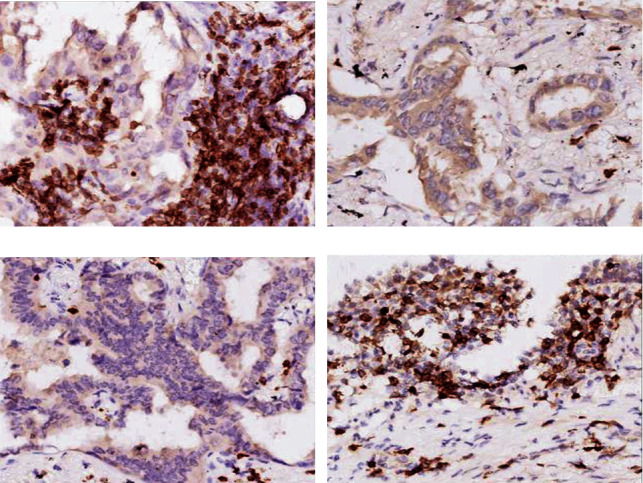
Expression of CD3 biomarker in the lung adenocarcinoma and adjacent normal tissues. Immunohistochemical staining of CD3 in the resected lung adenocarcinoma and adjacent normal lung tissues was performed as described in the methods. (a) Stage I lung adenocarcinoma. (b) Stage II lung adenocarcinoma. (c) Stage III lung adenocarcinoma. (d) Adjacent normal lung tissue. Magnification: 400x.

The expression of T lymphocyte subset biomarkers (CD3, CD4, and CD8) in the lung adenocarcinomas was significantly varied depending on the clinical stages. As shown in Figure 3, strong expression of CD3 biomarker was detected in the normal adjacent tissues as well as stage I tumor tissues, while it was significantly reduced in stage II and III lung adenocarcinomas. As shown in Table 3, strong expression of CD3 was detected in majority of stage I tumor tissues (87.5%, 35/40), while only 12.5% (5/40) of stage I tumors had mild expression of CD3, which was significantly different by chi-square comparison (*P* < 0.01). In contrast, weak expression of CD3 was noticed in majority of stage III tumor tissues (73.3%, 11/15), which was significantly higher compared to the percentage of stage III tumor with strong expression of CD3 (26.7%, 4/15, *P* < 0.01). There was no significant difference between the percentage of weak expression and strong expression of CD3 in the stage II tumor tissues (46.2% vs. 53.9%, *P* > 0.05).

**Figure 4:**
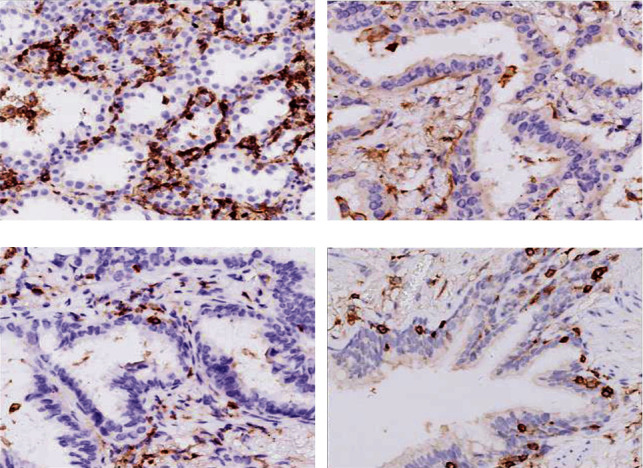
Expression of CD4 biomarker in the lung adenocarcinoma and adjacent normal tissues. Immunohistochemical staining of CD4 in the resected lung adenocarcinoma and adjacent normal lung tissues was performed as described in the methods. (a) Stage I lung adenocarcinoma. (b) Stage II lung adenocarcinoma. (c) Stage III lung adenocarcinoma. (d) Adjacent normal lung tissue. Magnification: 400x.

**Figure 5:**
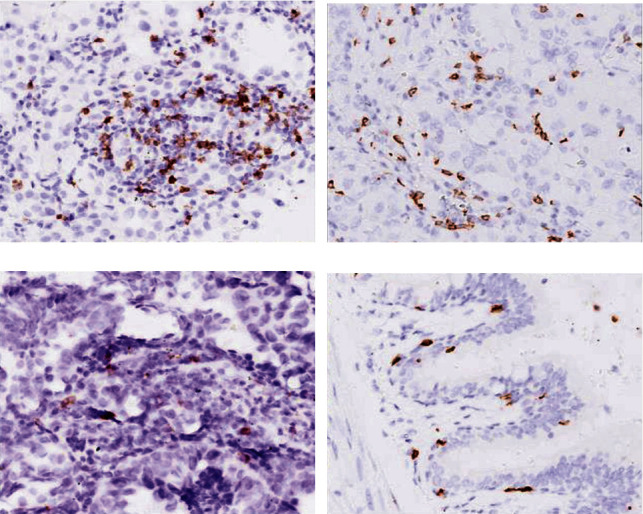
Expression of CD8 biomarker in the lung adenocarcinoma and adjacent normal tissues. Immunohistochemical staining of CD8 in the resected lung adenocarcinoma and adjacent normal lung tissues was performed as described in the methods. (a) Stage I lung adenocarcinoma. (b) Stage II lung adenocarcinoma. (c) Stage III lung adenocarcinoma. (d) Adjacent normal lung tissue. Magnification: 400x.

Similar to the CD3 biomarker, as shown in Figure 4, CD4 expression was detectable in the adjacent normal lung tissues (Figure 4(d)) and slightly higher in stage I lung adenocarcinomas (Figure 4(a)), but expression of CD4 was significantly reduced in stage II (Figure 4(b)) and III (Figure 4(c)) adenocarcinomas. Consistently, as shown in Table 4, strong expression of CD4 was detected in 90% (36/40) of stage I lung cancer tissues, which was significantly higher than the percentage of weak CD4 expression (10%, 4/40, *P* < 0.05). In contrast, in stage III tumor tissues, weakly expressed CD4 was noticed in majority of the samples (80%, 12/15), which was significantly higher than the percentage of samples with strong expression of CD4 (20%, 3/15, *P* < 0.01). Similarly, CD8 expression was also detected in the normal tissues (Figure 5(d)), and it was slightly higher in stage I lung adenocarcinomas (Figure 5(a)), but it was significantly reduced in stage III tumor tissues (Figure 5(c)). As shown Table 5, strong expression of CD8 was detected in majority of stage I tumor tissues (80%, 32/40), which was significantly different from the percentage of stage I tumors with weak expression of CD8 (20%, 8/40, *P* < 0.05). In stage III tumor tissues, however, majority tissues (86.7%, 13/15) had weak expression of CD8 biomarkers, which was significantly different from that of stage III tumors with strong expression of CD8 (13.3%, 2/15, *P* < 0.01).

## 4. Discussion

In this study, protein level and mRNA expression of IDO1 in the resected stage I, II, and III lung adenocarcinomas as well as adjacent normal lung tissues were assessed. It was found that IDO1 was significantly upregulated in stage I, II, and III lung adenocarcinomas compared to that of adjacent normal lung tissues. Furthermore, it was also found that the number of tumor-infiltrating CD3+, CD4+, and CD8+ lymphocytes was slightly increased in stage I lung adenocarcinomas compared to that in the adjacent normal lung tissues, but it was significantly reduced in stage II and III lung adenocarcinomas.

IDO1 is the rate-limiting enzyme in tryptophan catabolism, with limited expression in normal adult tissues (lymphoid tissues and placenta). However, IDO1 is highly expressed in variety of kinds of tumors including lung cancer [22–25]. IDO1 catalyzes tryptophan into kynurenine, resulting in tryptophan depletion and excessive accumulation of kynurenine, which induces apoptosis/dysfunction of CD4+ T cells and promotes regulatory T cell differentiation [22, 24, 26]. In addition, accumulation of toxic kynurenine metabolites can lead to activation of the amino acid-sensitive general control nondepressible 2 (GCN2) stress kinase pathway [16] and mammalian target of rapamycin (mTOR) pathway [27]. Activation of GCN2 pathways leads to cell cycle arrest and autophagy directly, which can exert cytostatic and cytotoxic effects on various immune cells, including CD8+ T lymphocytes, natural killer (NK) cells, and invariant NK-T cells [24, 28, 29]. In the current study, therefore, level of IDO1 expression and number of tumor-infiltrating T cell subsets were assessed in the lung adenocarcinoma and its adjacent normal tissues. We found that the later stage of lung adenocarcinoma was the higher level of IDO1 was expressed, suggesting IDO1 may play an important role in the progress of lung adenocarcinoma.

Studies have shown that IDO1 exerted a powerful immunosuppressive effect through local tumor microenviroment inhibition of T lymphocytes and other immune cells [23, 30, 31]. Further studies have shown that the expression of IDO1 was correlated with the expression of CD4, CD3, and CD8 as well as prognosis in colon cancer [32], but the expression of IDO1 was not related to the expression of CD3 and CD8 in the hormone receptor-positive breast cancer [33]. In lung cancer patients, previous studies reported that high expression of IDO1 was associated with poor prognosis and survival in non-small-cell lung cancer [21, 34], and IDO1 activity was linked to primary resistance to immunotherapy in non-small-cell lung cancer [25]. In this study, we found that all three subsets of T lymphocytes (CD3+, CD4+, and CD8+) infiltrated into the lung adenocarcinoma as well as adjacent normal lung tissues. However, tumor infiltration of T lymphocytes varied along the development of the lung adenocarcinoma from stages I through III, that is, the T cell infiltration was increased at the early stage of the cancer (stage I), but it was gradually decreased at middle (stage II) and late (stage III) stages, while expression of IDO1 gradually increased from early to later stage lung adenocarcinoma, suggesting IDO1 may contribute to the inhibition on the tumor infiltration of T cells in the lung adenocarcinoma.

There were limitations in the current study. First, this was a single-center cohort study with small sample size, and thus, findings of this study remain to be further confirmed in a large scale and multicenter cohort study. Second, mechanisms of IDO1 suppression on T cell infiltration and survival/apoptosis in the tumors were not explored in this study. Third, we expected IDO1 expression was further increased while the number of T cells was further reduced in stage IV lung adenocarcinoma tissues. However, due to unfavored clinical outcome, stage IV lung adenocarcinoma patients were not enrolled into this study, which could weaken the findings of this study. Fourth, correlation of IDO1 expression and patients' survival and clinical outcomes including quality of life remains to be analyzed in the future study.

Taken together, expression of IDO1 and tumor infiltration of T cell subsets in various clinical stages of lung adenocarcinomas as well as adjacent normal lung tissues were investigated in this study. It was found that the later clinical stage of lung adenocarcinoma was, the higher expression of IDO1 in the tumor but the less infiltration of T lymphocytes was found in the lung adenocarcinomas. These findings indicated that IDO1 may contribute to the immune escape of tumor cells in lung adenocarcinoma through suppression on the infiltration and survival of T cells in the tumor microenvironment. Level of IDO1 expression in combination with the clinical stage of lung adenocarcinoma could be used to evaluate the feasibility of radical surgery of the lung cancer.

## Figures and Tables

**Table 1:** Demographic information of the participants.

Clinical parameters	Patients cohort	
	*N*	%
Gender		
Male	39	57.35
Female	29	42.65
Age		
<68 years	52	76.47
≥68 years	16	23.53
Smoking		
Current smokers	18	26.47
Ex-smokers	5	7.35
Never smokers	45	66.18
Histology		
Adenocarcinoma	68	100
Cancer stages		
Stage I	40	58.82
Stage II	13	19.12
Stage III	15	22.06

**Table 2:** Expression level of IDO1 and cancer stages.

Stages	IDO1 low		IDO1 high		
	*N*	%	*N*	%	*P* ^∗^
I	37	92.5	3	7.5	*P*1 < 0.01
II	4	30.8	9	69.2	*P*2 > 0.05
III	1	6.7	14	93.3	*P*3 < 0.01
Subtotal	42	61.8	26	38.2	

^∗^Compared by Pearson's *χ*^2^ test.

**Table 3:** Tumor infiltration of CD3+ T cells in the three stages of lung adenocarcinomas.

Stages	Low CD3+ cells		High CD3+ cells		
	*N*	%	*N*	%	*P* ^∗^
I	5	12.5	35	87.5	*P*1 < 0.01
II	6	46.2	7	53.9	*P*2 > 0.05
III	11	73.3	4	26.7	*P*3 < 0.01
Subtotal	22	32.4	46	67.6	

^∗^Compared by Pearson's *χ*^2^ test.

**Table 4:** Tumor infiltration of CD4+ T cells in the three stages of lung adenocarcinomas.

Stages	Low CD4+ cells		High CD4+ cells		
	*N*	%	*N*	%	*P* ^∗^
I	4	10.0	36	90.0	*P*1 < 0.01
II	7	53.9	7	46.2	*P*2 > 0.05
III	12	80.0	3	20.0	*P*3 < 0.01
Subtotal	23	33.8	46	67.6	

^∗^Compared by Pearson's *χ*^2^ test.

**Table 5:** Tumor infiltration of CD8+ T cells in the three stages of lung adenocarcinomas.

Stages	Low CD8+ cells		High CD8+ cells		
	*N*	%	*N*	%	*P* ^∗^
I	8	20.0	32	80.0	*P*1 < 0.01
II	11	84.6	2	15.4	*P*2 > 0.05
III	13	86.7	2	13.3	*P*3 < 0.01
Subtotal	32	47.1	36	52.9	

^∗^Compared by Pearson's *χ*^2^ test.

## Data Availability

The datasets generated and analyzed during the current study are available from the corresponding author on reasonable request.

## Abbreviations

ALK: Anaplastic lymphoma kinase
ADC: Adenocarcinoma
EGFR: Growth factor receptor
FFPE: Formalin-fixed, paraffin-embedded
mTOR: Mammalian target of rapamycin
GCN2: General control nondepressible 2
IDO1: Indoleamine 2,3-dioxygenase 1
Kyn: Kynurenine
LCC: Large cell carcinoma
NSCLC: Non-small-cell lung cancer
NK: Natural killer
PD-1: Programmed cell death-1
PD-L1: Programmed cell death-ligand 1
SCLC: Small-cell lung cancer
SQC: Squamous cell carcinoma
Treg: Regulatory T cells.
